# Antibiotic-Resistant *Salmonella* Enteritidis in Raw Chicken Meat of Dhaka City, Bangladesh

**DOI:** 10.1155/ijm/5654730

**Published:** 2025-03-23

**Authors:** Shah Jungy Ibna Karim, K. B. M. Saiful Islam, M. Rubaiyat Adnan, Md. Abir Hassan Sadi, Mahfuzul Islam

**Affiliations:** ^1^Department of Medicine and Public Health, Sher-e-Bangla Agricultural University, Dhaka, Dhaka Division, Bangladesh; ^2^Department of Microbiology and Parasitology, Sher-e-Bangla Agricultural University, Dhaka, Dhaka Division, Bangladesh

**Keywords:** antimicrobial resistance, *Inv*A gene, multiple antibiotic resistance index, raw chicken meat, *Salmonella* Enteritidis, *Tet*A gene

## Abstract

Foodborne zoonotic *Salmonella* is transmitted through contaminated meat, milk, and eggs. This study is aimed at investigating the antimicrobial resistance (AMR) profile of *Salmonella* Enteritidis isolated from raw chicken meat in Dhaka City, Bangladesh. Two hundred raw chicken meat samples were aseptically collected from 20 retail markets located in Dhaka City, and the isolated *Salmonella* species were identified based on their morphological, cultural, biochemical, and molecular characterization by polymerase chain reaction (PCR). The primer sets of the *Inv*A and Enteritidis-specific STM3098 gene were used for the PCR detection of *Salmonella* species and *S.* Enteritidis, respectively. The isolates were then screened for AMR phenotypically and the presence of the tetracycline resistance (*Tet*A) gene. The prevalence of *Salmonella* species and *S.* Enteritidis was 22.5% (*n* = 45/200) and 18.5% (*n* = 37/200), respectively. However, the prevalence was constant across all the sample markets (*p* > 0.05). Tetracycline, amoxicillin, and ampicillin resistance was phenotypically present in all isolates (100%). Furthermore, approximately 70%, 49%, and 30% of *S.* Enteritidis showed resistance against erythromycin, amoxicillin/clavulanic acid, and ciprofloxacin, respectively. However, *S.* Enteritidis were sensitive to gentamicin (86.5%), meropenem (64.9%), ciprofloxacin (62.2%), and ceftriaxone (59.5%). The *Tet*A gene, which causes AMR against tetracycline, was shown to be present in all phenotypically resistant *Salmonella* species. Multiple antibiotic resistance index (MARI) ranged between 0.3 and 0.8. Overall, multidrug resistant (MDR) *Salmonella* Enteritidis emerged in the chicken meat along with the presence of a resistance gene that is a threat to human health. Therefore, action must be taken to stop the spread of AMR.

## 1. Introduction

In many parts of the world, meat makes up a considerable portion of a typical diet by contributing protein, minerals, vitamins, and fat. Among meat, chicken meat is a readily available and affordable high-quality animal protein in Bangladesh, and the demand of it has increased due to the extensive development of the poultry industry in Bangladesh. However, it can be contaminated with microorganisms, especially zoonotic *Salmonella*, which can be transmitted to humans through the food chain.

Foodborne illness in human occurs due to the consumption of a particular food or drink contaminated with various pathogenic microbes [1]. Due to inadequate or nonexistent reporting systems, most developing nations have a scarcity of accurate statistics on foodborne infections. Foodborne illnesses are mostly caused by bacteria, viruses, and parasites [1].

*Salmonella* is one of the more prevalent causes of foodborne diarrheal disorders across the world, and many of these infections are zoonotic, meaning they are spread from seemingly healthy carrier animals to people via contaminated foods. Food animals are the principal reservoirs of zoonotic *Salmonella*, and animal-derived items such as meat, milk, eggs, and their products are the main causes of infections in industrialized nations [2]. Contaminated water, food, and human-to-human transmission, on the other hand, lead to a disproportionately higher number of human cases in impoverished nations than in developed countries [3]. Globally, one of the main causes of enteric illnesses is *Salmonella enterica*. It results in a significant number of illnesses and financial losses for both developed and developing nations. It is frequently linked to human gastroenteritis and foodborne salmonellosis [4, 5].

The main factor causing pathogens to develop drug resistance is the overuse and abuse of antibiotics in both humans and animals [2]. Since antibiotic resistance can spread to other bacteria, food contaminated with drug-resistant bacteria poses a serious risk to public health [6]. Furthermore, the multiple antibiotic resistance index (MARI) indicates the level of risk in the environment where antibiotics are often used [7]. A previous study revealed the phenotypic antibiotic resistance pattern of *Salmonella* spp. in poultry meat in Dhaka City, Bangladesh [8]; however, little is known about the genotypic resistance on species level. Additionally, Dhaka City was chosen as the study area because it is a populated city in Bangladesh, where chickens from all over the nation congregate and the pathogen burden is likely to increase. One of the major global health risks associated with antibiotic-resistant pathogens is *Salmonella* Enteritidis. So, the present study was taken to investigate the antimicrobial resistance profile of *S.* Enteritidis isolated from raw chicken meat of Dhaka City, Bangladesh.

## 2. Material and Methods

### 2.1. Ethical Approval and Informed Consent

Ethics committee approval is not needed for this kind of research. In the wet market, chicken was sold commercially for human consumption.

### 2.2. Study Period, Area, and Collection of Samples

From July 2022 to June 2023, the entire study was carried out in the laboratory of the Department of Microbiology and Parasitology at Sher-e-Bangla Agricultural University (SAU), Dhaka-1207. We used sterile polythene bags to gather 200 raw chicken meat samples from 20 randomly chosen retail markets in Dhaka City. Afterwards, using an ice box, the obtained meat samples were moved to the respective laboratory.

### 2.3. Isolation and Tentative Identification

*Salmonella* spp. were isolated and characterized as in earlier reports [9, 10]. For *Salmonella* isolation, enrichment of samples was performed in selenite cystine broth and then, samples were cultured onto Salmonella–Shigella (SS) agar. To get pure cultures, subculture technique was followed. The isolated pure colonies were preserved in 20% sterile buffered glycerin and on nutrient agar slants for further analysis [11]. For observing the additional cultural properties, MacConkey (MC) agar and xylose–lysine–deoxycholate (XLD) agar were used. Motility was checked by the hanging drop technique [11]. For biochemical characterization, catalase, carbohydrate fermentation, methyl red (MR), Voges–Proskauer (VP), and indole tests and the triple sugar iron agar slant reaction were performed [11].

### 2.4. DNA Extraction

Total genomic DNA was recovered from *Salmonella* cells using the traditional boiling procedure [12]. After transferring 1 mL of the cultured material into a 1.5-mL Eppendorf tube, it was centrifuged for 5 min at 14,000 × *g*. To create a larger particle, 1 mL of culture was added to the pellet and centrifuged again after the supernatant was disposed of. After adding 600 *μ*L of sterile distilled water to the pellet, it was centrifuged at 14,000 × *g* for 5 min. The supernatant was disposed of, and 200 *μ*L of sterile distilled water was added again. The mixture was then heated to 100°C (Labnet, Florida, United States) for 10 min, followed by an immediate 5 min of ice cooling. Following cooling, the material was centrifuged for 5 min at 14,000 × *g*. The resultant supernatant was put into a fresh Eppendorf tube and kept at −20°C until the PCR was performed.

### 2.5. Polymerase Chain Reaction (PCR) to Identify *Salmonella* spp.

The presence of *invA* and STM3098 genes was tested to identify *Salmonella* spp. and *S.* Enteritidis. A single reaction of 25 *μ*L including one Ex Taq buffer (Mg2+ plus), 0.4 *μ*M of each primer, 200 *μ*M of dNTP, 0.5 U of Ex Taq DNA polymerase, and 25 ng/*μ*L of template DNA from the isolated Salmonella serovars. An initial heating at 94°C for 1 min was followed by 30 cycles of denaturation at 94°C for 1 min, annealing at 64°C for 30 s, extension at 72°C for 30 s, and concluding with a final extension at 72°C for 7 min in a thermocycler (GeneAtlas, Model G02, Japan). The amplified products were subjected to electrophoresis on a 1.5% agarose gel. A 50-bp DNA ladder was used as a standard marker.

### 2.6. Antibiotic Sensitivity Tests of Isolated *Salmonella*

Using the Kirby–Bauer disk diffusion method, the identified *Salmonella* species were tested for antimicrobial sensitivity against ten widely used antibiotics of various classes [13]. Briefly, bacterial inoculums (grown overnight and adjusted to a 0.5 McFarland standard) were swabbed with a sterile cotton swab and allowed to dry for 10–15 min on Mueller–Hinton agar (MHA) plates. After sterile forceps were used to place antibiotic discs (Oxoid Ltd., United Kingdom) on MHA plates, an aerobic 24-h incubation period was carried out at 37°C. CLSI standards [14] were followed in classifying the organisms as “resistant” or “intermediate” or “susceptible” based on the diameter of their zone of inhibition after incubation. The following substances are among the antibiotic classes and antibiotics used in this investigation: ampicillin (AMP), 10 *μ*g; amoxicillin (AMX), 30 *μ*g; amoxicillin–clavulanic acid (AMC), 20/10 *μ*g; ceftriaxone (CTR), 30 *μ*g; ciprofloxacin (CIP), 5 *μ*g; nalidixic acid (NA), 30 *μ*g; erythromycin (E), 15 *μ*g; gentamicin (GEN), 10 *μ*g; tetracycline (TE), 30 *μ*g; and meropenem (MEM), 10 *μ*g. “Multidrug resistance” (MDR) refers to the resistance of Enterobacteriaceae, which includes *Salmonella* spp., to at least one agent from three or more of classes of antibiotics [15]. Using the procedure outlined by Ateba and Bezuidenhout [16], multiple antibiotic resistance (MAR) patterns were produced for each isolate.

### 2.7. Multiple Antibiotic Resistance Index (MARI)

The following formula was used to calculate and interpret the isolates' MARI in accordance with Krumperman's [17] methodology: MARI = *a*/*b*, where “*b*” is the total number of antibiotics tested and “*a*” is the number of antibiotics to which a specific isolate was resistant. According to Osundiya et al. [7], a MARI of ≥ 0.2 denotes a high-risk setting where antibiotics are frequently used.

### 2.8. PCR to Identify *tet*A-Resistant Gene

The PCR process was carried out using 50 *μ*L (total volume) of distilled H_2_O, 0.25 mM of each deoxyribonucleotide, 1.5 mM MgCl_2_, 0.2 U of Gold Taq DNA polymerase, and 50 pmol of each primer. An initial template denaturation step of 95°C for 1 min was included in the temperature profile. This was followed by 30 cycles of 95°C for 30 s, 55°C for 1 min, 72°C for 1 min, and a final step of 72°C for 7 min (GeneAtlas, Model G02, Japan). The amplified products were subjected to electrophoresis on a 1.5% agarose gel. A 50-bp DNA ladder was used as a standard marker.

### 2.9. Molecular Detection of *Salmonella* spp.

All the *Salmonella* isolates and resistance pattern were confirmed through molecular detection (see primers in Table 1).

### 2.10. Statistical Analysis

Minitab 17 (Minitab Ltd., United Kingdom) was used to analyze the study's data. Pearson's chi-square test was used to determine any significant differences between the variables.

## 3. Results

### 3.1. Identification of *Salmonella* spp.

**Figure 1:**
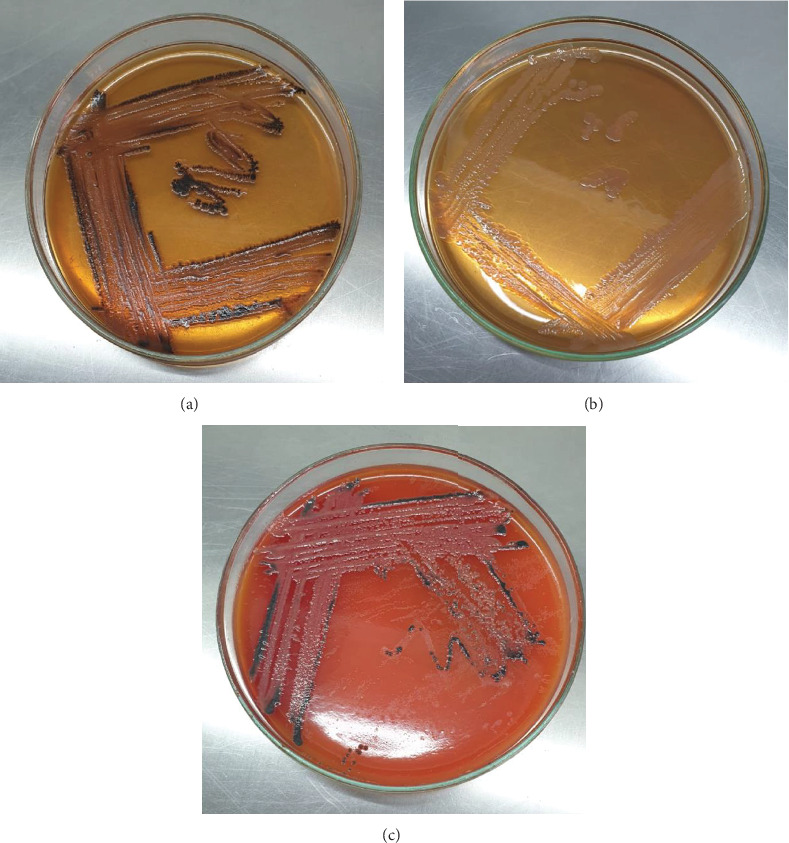
Cultural properties of the isolated *Salmonella* species by detecting (a) colorless black centered colonies on SS agar, (b) colorless non–lactose fermenter colonies on MC agar; (c) black centered nonfermented colonies with slightly red–colored translucent zone on XLD agar.

*Salmonella* species formed colorless black centered colonies on the S-S agar, appeared to be colorless nonlactose fermenter colonies on MC agar, and formed black centered nonfermented colonies with a slightly red–colored translucent zone on XLD agar (Figure 1). *Salmonella* species exhibited gram-negative short rods or coccobacilli organized in pairs or in single forms in accordance with Gram staining (Table 2). *Salmonella* spp. fermented dextrose, maltose, and mannitol along with the production of acid and gas, according to the carbohydrate fermentation test; however, these bacteria did not ferment lactose or sucrose (Table 2). The indole and VP tests were negative, while the methyl red and catalase test showed a positive result (Table 2).

### 3.2. Molecular Identification of *Salmonella* spp. and *Salmonella* Enteritidis

**Figure 2:**
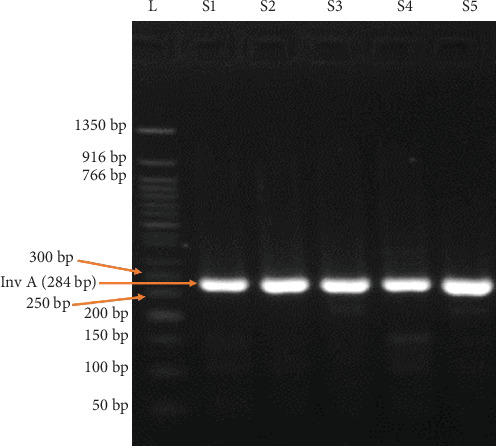
PCR confirmed *Salmonella* species by detecting the InvA gene. L = ladder; S1–S5 = Samples 1–5 (representative samples).

**Figure 3:**
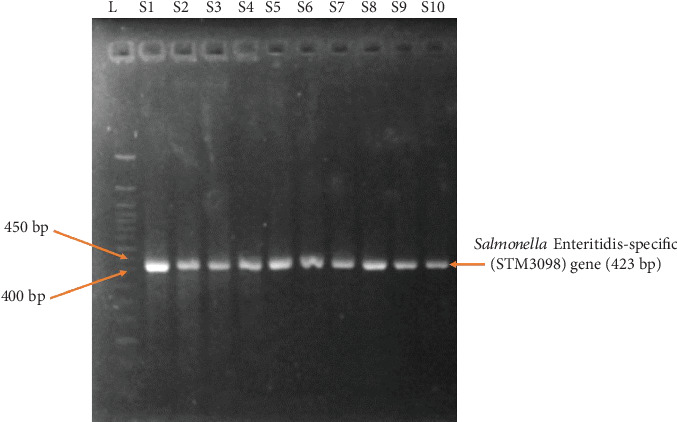
PCR confirmed *S.* Enteritidis by detecting Enteritidis-specific (STM3098) gene. L = ladder; S1–S10 = Samples 1–10 (representative samples).

DNA isolated from 45 *Salmonella* samples were subjected to PCR amplification. The 284-bp fragment of invasion gene (Figure 2) and 423-bp fragment of Enteritidis*-*specific STM3098 gene were confirmed for all isolates (Figure 3).

### 3.3. Prevalence of *Salmonella* Species in Raw Chicken Meat

According to the data in Table 3, 45 (22.5%) and 37 (18.5%) samples had positive results for *Salmonella* species and *Salmonella* Enteritidis, respectively. However, the prevalence was consistent throughout all sampling markets (*p* > 0.05).

### 3.4. Antibiotic-Resistant Pattern of *Salmonella* Species Isolated From Raw Chicken Meats

**Figure 4:**
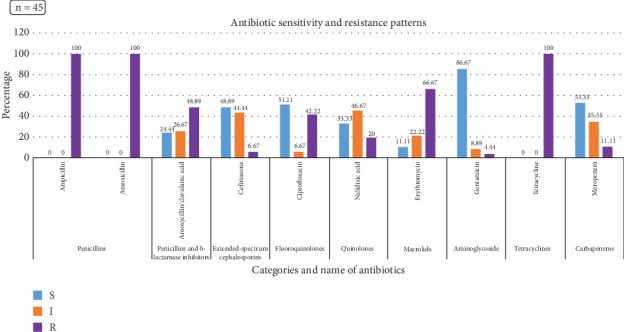
*Salmonella* species with antibiotic resistance patterns found in raw chicken meat from Dhaka City. S = sensitive, I = intermediate, R = resistant.

**Figure 5:**
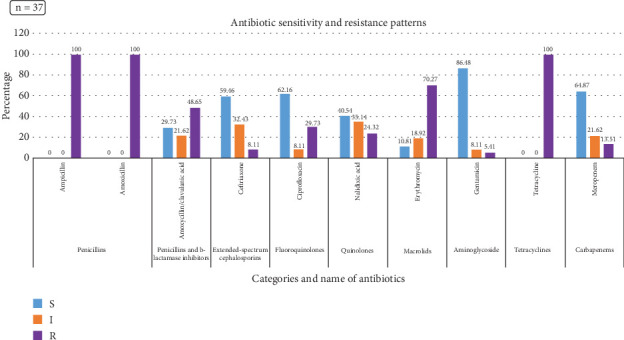
*Salmonella* Enteritidis with antibiotic resistance patterns found in raw chicken meat from Dhaka City. S = sensitive, I = intermediate, R = resistant.

In the current phenotypic investigation, all *Salmonella* species showed 100% resistance against TE, AMX, and AMP followed by approximately 67%, 49%, and 42% against E, AMC, and CIP, respectively (Figure 4). Similarly, 100% of the *S.* Enteritidis were phenotypically resistant against TE, AMX, and AMP, which was followed by 70%, 49%, and 30% for E, AMC, and CIP, respectively (Figure 5). However, approximately 87%, 53%, 51%, and 49% of the *Salmonella* species showed susceptibility to GEN, MEM, CIP, and CTR, respectively. Likewise, *S.* Enteritidis isolates were susceptible to GEN (86.5%), MEM (64.9%), CIP (62.2%), and CTR (59.5%).

### 3.5. MDR Bacteria

All isolates of *Salmonella* and *S.* Enteritidis (100%) isolates were resistant to at least three of the antibiotics used in this study. Microorganisms that showed resistance to at least one agent in three or more antibiotics classes were considered MDR (Tables 4 and 5). Among 45 *Salmonella* isolates, 18, 12, 9, and 4 isolates were resistant to three, four, five, and six different classes of antibiotics. Only one *Salmonella* isolate was resistant to seven different classes of antibiotics. *S.* Enteritidis isolates were resistant to three, four, five, and six classes of antibiotics according to 14, 12, 5, and 5 in numbers.

### 3.6. MAR Phenotypes and MARIs

The MAR pattern of each isolate is displayed in Table 6, and nearly all of the isolates (36 out of 37) were resistant to more than four antibiotics tested. A total of 2.7% (*n* = 1) of the *S.* Enteritidis showed MAR phenotype against eight antibiotics, whereas 37.8% (*n* = 14), 32.4% (*n* = 12), 13.5% (*n* = 5), and 10.8% (*n* = 4) of the isolates showed the MAR phenotype against four, five, six, and seven antibiotics, respectively. The MARI ranged between 0.3 and 0.8.

### 3.7. Prevalence of Resistance Genes

**Figure 6:**
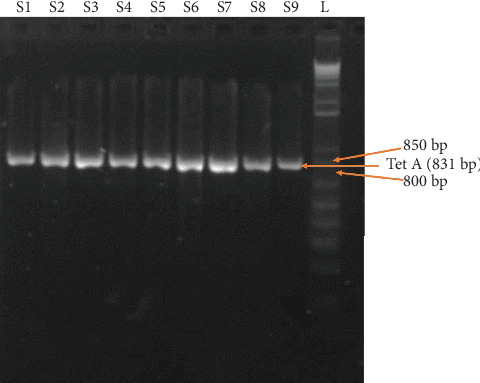
PCR detected the *tet*A gene among the *Salmonella* species. L = ladder; S1–S9 = Samples 1–9 (representative samples).

In this study, all the phenotypically resistant *Salmonella* species were positive for the *Tet*A gene, responsible for antimicrobial resistance against TE. Representative sample (Figure 6 and Table 7) and primers are presented in Table 1.

## 4. Discussion

Pathogenic MDR *Salmonella enterica* serovars are a global public health concern and a major source of foodborne illnesses. *Salmonella* is one of the leading causes of foodborne illnesses worldwide [21]. Every year, 420,000 people die from foodborne illnesses, and one in 10 people become infected [22]. Using the *Inv*A and Enteritidis-specific STM3098 gene from a previous work employing a similar primer, Kaushik, Anjay, and Dayal [18] and Kim et al., [19], the presence of *Salmonella* and *S.* Enteritidis was identified. Out of 200 samples, 45 (22.5%) tested positive for *Salmonella* spp. and 37 (18.5%) tested positive for *S.* Enteritidis in the current investigation. The prevalence rate of *Salmonella* in the chicken value chain was reported by Al Mamun et al. [23] to be 23.53%, which is fairly similar to the results of the current study. In two previous studies from Bangladesh, Mahmud et al. [24] and Rabby et al. [8], respectively, revealed higher (37.9%) and lower (13.46%) *Salmonella* prevalence rates. Al-Salauddin et al. [25] also reported that a small number of isolated locations in Bangladesh accounted for 31.66% of samples that tested positive for *Salmonella* spp. According to both the current study and the earlier report, eating chicken meat tainted with *S.* Enteritidis increases the risk of human foodborne illness in Bangladesh. Numerous nations have also reported cases of *Salmonella* contamination in chicken meat. Turkey's Arkali and Çetinkaya [26] discovered that 21.9% of *S.* Enteritidis from raw chicken had comparable outcomes. In contrast, the current study discovered a greater incidence of *S.* Enteritidis in chicken and beef that were gathered from retail markets in Malaysia (6.70% and 1.25%, respectively) than prior studies carried out by Thung et al. [27] and Thung et al. [28]. The use of antibiotics and management techniques in chicken farms, the cleanliness of slaughterhouses, the water used for dressing, the study's time period, geographic location, etc. could all contribute to the variation in *Salmonella* sp. prevalence across studies.

Our study's findings indicate that AMX, AMP, TE, and E are often antibiotics utilized in Bangladesh's chicken farming system due to the high resistance of *Salmonella* spp. and *S*. Enteritidis to these drugs. The results of this study are in line with those of Alam et al. [29], who found that isolates of *Salmonella* were resistant to TE, AMP, streptomycin, and chloramphenicol in a range of 77.1%–97.1%. Likewise, Bupasha et al. [30] found that *Salmonella* found in pigeons had resistance rates to AMX, E, and TE of 93.1%, 81.8%, and 86.2%, respectively. Higher resistance to E, AMP, AMX, and TE was also discovered by Karim et al. [31]. According to Rabby et al. [8], all discovered Salmonella species showed resistance to E, AMP, cloxacillin, trimethoprim, and nitrofurantoin. Minami et al. [32] report that most isolates of *Salmonella* show resistance to both streptomycin and TE. Furthermore, several *Salmonella* strains that were resistant to AMP, GEN, chloramphenicol, and kanamycin were reported to be MDR. Due to the extensive use of antibiotics in animals, many bacterial strains that pose a risk to humans through the food chain have grown more resistant to them according to Thai et al. [2]. On the other hand, in contrast to the findings of Siddiky et al. [33] and Siddiky et al. [34], the isolates of *Salmonella* and *S.* Enteritidis showed lower levels of resistance to CIP. This discrepancy could be caused by the study's smaller sample size than in the other research.

Every isolate of *Salmonella* and *S.* Enteritidis in our investigation (100%) exhibited resistance to a minimum of three antibiotics. The results of Siddiky et al. [33], Siddiky et al. [34], and Karim et al. [31] exhibit MDR patterns. Mishra et al. [35] stated that a MARI of 0.2 or higher denotes high-risk sources of contamination, whereas a MARI of 0.4 or higher is linked to sources of contamination that are fecal. According to Thenmozhi et al. [36], MARI values greater than 0.2 signify the presence of an isolate from a high-risk contaminated source that uses antibiotics frequently, whereas values less than 0.2 suggest germs from a source that uses antibiotics less frequently. Vigilant surveillance and corrective action are required in cases of high MARI. Out of the 10 antibiotics utilized in our investigation, the MAR phenotype was found for three to eight antibiotics, and the MARI varied from 0.3 to 0.8, which is consistent with the results of investigations by Hossain et al. [37] using commercial eggs and is further corroborated by other research projects [29, 38, 39].

It is noted that tetA is one of the important genes present in *Salmonella* which is responsible for the development of resistance against tetracycline [40]. In this study, all isolated *Salmonella* species and *S.* Enteritidis (100%) that showed resistance to TE were found to carry the TetA gene. The resistance patterns observed were consistent with previous findings, both domestically and internationally. The most common occurrence of *tetA* in broiler chickens in Iran was identified by Jahantigh et al. [41]. TetA and blaTEM were the most frequently found genes in nontyphoidal *Salmonella* isolated from Indian retail chicken stores, according to Sharma et al. [42]. Siddiky et al. [34] found *tetA*, *sul1*, and *strA/B* in chicken cecal content of Bangladesh, while Alam et al. [29] found the presence of *tetA* (97.14%) and blaTEM-1 (82.9%) genes in broiler farms.

Our findings indicated that wet markets, where chickens are slaughtered, processed, and sold, have the potential to spread *Salmonella* spp. and *S.* Enteritidis. Several studies have shown that poor sanitary and hygienic practices may cause cross-contamination of poultry during processing and skinning in wet markets [43]. Nidaullah et al. [43] added that vendors, swabs from chopping boards and knives, carcass dressing water, defeathering machines, scalding water, tanks, floors, drains, and workbenches are possible sources of contamination in wet markets. The existence of free-roaming MDR serovars in poultry processing environments may facilitate their introduction through wet markets into the food chain, agricultural products, and human populations. Additionally, it was found that *Salmonella* serovars can survive for extended periods in soil-formed biofilms, which offer protection against detergents and sanitizers according to Chmielewski and Frank [44]. Therefore, in order to reduce the possibility of *Salmonella enterica* spreading horizontally in wet markets, it is imperative to stress the importance of regularly changing the carcass dressing water and cleaning and sterilizing knives and chopping boards. The underlying reasons suggest that the emergence of MDR serovars in poultry is linked to the inappropriate use of antimicrobials in farming practices.

## 5. Conclusion

The identification of highly concerning pathogenic MDR *Salmonella enterica* serovar Enteritidis in the chicken meat obtained from retail wet markets has raised serious concerns for public health. Commercial chicken harbor pathogenic MDR strains of *Salmonella enterica*. The outcome showed that wet markets where chicken carcasses are processed are harbors for *Salmonella enterica* serovars. Due to inadequate hygiene practices, the wet market may be regarded as a hotspot for the transmission and contamination of MDR *Salmonella enterica* serovars, which can quickly attach themselves to vendors, customers, and the food chain. It is also recommend that consumers should avoid contamination of food that is not cooked, by using separate cutting boards and by hand sanitation. Furthermore, the inappropriate use of antibiotics in the production cycle or environmental spillover may be the cause of resistance in healthy chicken. The results of this study will help create and implement a national AMR surveillance program that will ensure food safety and market control to further slow the spread at wet markets. More resistance patterns would alert lawmakers, scientists, and medical professionals to the need for standard treatment protocols. They would promote adopting suitable agricultural practices for the prudent and responsible use of antibiotics in the chicken production system.

## Figures and Tables

**Table 1:** Oligonucleotide primers used in this study.

**Target genes**	**Primers sequence (5**⁣′**-3**⁣′**)**	**Amplicon size (bp)**	**References**
*Inv*A	F: GTGAAATTATCGCCACGTTCGGGCAA R: TCATCGCACCGTCAAAGGAACC	284	Kaushik, Anjay, and Dayal [18]
Enteritidis-specific STM3098 gene	F: TTTGGCGGCGCAGGCGATTC R: GCCTCCGCCTCATCAATCCG	423	Kim et al. [19]
*tet*A	F: GCGCCTTTCCTTTGG GTTCT R: CCACCCGTTCCACGT TGTTA	831	Chen et al. [20]

**Table 2:** Staining, motility, and biochemical properties of the isolated *Salmonella* spp.

**Parameters**	**Properties**
Gram staining	Gram-negative coccobacilli
Motility	Motile
Sugar fermentation	
Dextrose	AG
Lactose	−ve
Maltose	AG
Sucrose	−ve
Mannitol	AG
MR	+ve
VP	−ve
Indole	−ve
Catalase	+ve
Triple sugar iron agar slant reaction	Pinkish slant with production of H_2_S

*Note:* +ve = positive; −ve = negative; A = acid production; AG = acid and gas production.

Abbreviations: H_2_S = hydrogen sulfide, MR = methyl red, VP = Voges-Proskauer.

**Table 3:** Prevalence of *Salmonella* species and *Salmonella* Enteritidis isolated from raw chicken meat.

**Name of sampling market**	**No. of samples examined**	**Isolated *Salmonella* species**		**p** ** value (** **χ** ^2^ ** test)**	**Isolated *Salmonella* Enteritidis**		**p** ** value (** **χ** ^2^ ** test)**
		**No.**	**%**		**No.**	**%**	
Market 1	10	2	20	0.995	2	20	0.999
Market 2	10	3	30		2	20	
Market 3	10	3	30		3	30	
Market 4	10	2	20		2	20	
Market 5	10	3	30		1	10	
Market 6	10	2	20		2	20	
Market 7	10	3	30		1	10	
Market 8	10	2	20		2	20	
Market 9	10	1	10		1	10	
Market 10	10	2	20		2	20	
Market 11	10	3	30		1	10	
Market 12	10	2	20		2	20	
Market 13	10	3	30		3	30	
Market 14	10	3	30		3	30	
Market 15	10	1	10		1	10	
Market 16	10	1	10		1	10	
Market 17	10	3	30		2	20	
Market 18	10	2	20		2	20	
Market 19	10	3	30		3	30	
Market 20	10	1	10		1	10	
Total	200	45	22.5	—	37	18.5	—

*Note:p* value less than 0.05 was considered significant.

**Table 4:** Multidrug-resistant *Salmonella* spp. (*n* = 45) isolated from raw chicken meat.

**Antimicrobial compound**	**Antibiotic class**	**Number of MDR isolates (%)**
AMP-AMX-TE	Pen-Tet	1 (2.2)
AMP-AMX-TE-CIP	Pen-Tet-Flu	6 (13.3)
AMP-AMX-TE-GEN	Pen-Tet-Ami	1 (2.2)
AMP-AMX-TE-E	Pen-Tet-Mac	9 (20.0)
AMP-AMX-TE-NA	Pen-Tet-Qui	2 (4.4)
AMP-AMX-TET-AMC-E	Pen-Tet-PenB-Mac	5 (11.1)
AMP-AMX-TE-NA-E	Pen-Tet-Qui-Mac	2 (4.4)
AMP-AMX-TE-AMC-NA	Pen-Tet-PenB-Qui	2 (4.4)
AMP-AMX-TE-AMC-CTR	Pen-Tet-PenB-Cef	1 (2.2)
AMP-AMX-TE-CIP-NA	Pen-Tet-Flu-Qui	1 (2.2)
AMP-AMX-TE-E-MEM	Pen-Tet-Mac-Car	1 (2.2)
AMP-AMX-TE-AMC-CIP-E	Pen-Tet-PenB-Flu-Mac	7 (15.6)
AMP-AMX-TE-AMC-CTR-E	Pen-Tet-PenB-Cef-Mac	1 (2.2)
AMP-AMX-TE-AMC-NA-E	Pen-Tet-PenB-Qui-Mac	1 (2.2)
AMP-AMX-TE-AMC-CIP-E-MEM	Pen-Tet-PenB-Flu-Mac-Car	2 (4.4)
AMP-AMX-TE-AMC-CIP-NA-MEM	Pen-Tet-PenB-Flu-Qui-Car	1 (2.2)
AMP-AMX-TE-AMC-CIP-E-GEN	Pen-Tet-PenB-Flu-Mac-Ami	1 (2.2)
AMP-AMX-TE-AMC-CTR-CIP-E-MEM	Pen-Tet-PenB-Cef-Flu-Mac-Car	1 (2.2)
Total		45 (100.0)

Abbreviations: AMC = amoxycillin/clavulanic acid, Ami = aminoglycoside, AMP = ampicillin, AMX = amoxicillin, Car = carbapenem, Cef = cephalosporins, CIP = ciprofloxacin, CTR = ceftriaxone, E = erythromycin, Flu = fluoroquinolone, GEN = gentamicin, Mac = macrolide, MDR = multidrug resistant, MEM = meropenem, NA = nalidixic acid, pen = penicillin, PenB = penicillin–beta-lactamase inhibitor, Qui = quinolone, Tet = tetracycline.

**Table 5:** Multidrug-resistant *Salmonella* Enteritidis (*n* = 37) isolated from raw chicken meat.

**Antimicrobial compound** ^**a**^	**Antibiotic class** ^**a**^	**Number of MDR isolates (%)**
AMP-AMX-TE	Pen-Tet	1 (2.7)
AMP-AMX-TE-CIP	Pen-Tet-Flu	2 (5.4)
AMP-AMX-TE-NA	Pen-Tet-Qui	2 (5.4)
AMP-AMX-TE-GEN	Pen-Tet-Ami	1 (2.7)
AMP-AMX-TE-E	Pen-Tet-Mac	9 (24.3)
AMP-AMX-TET-AMC-E	Pen-Tet-PenB-Mac	5 (13.5)
AMP-AMX-TE-AMC-NA	Pen-Tet- PenB -Qui	2 (5.4)
AMP-AMX-TE-NA-E	Pen-Tet-Qui-Mac	2 (5.4)
AMP-AMX-TE-CIP-NA	Pen-Tet-Flu-Qui	1 (2.7)
AMP-AMX-TE-E-MEM	Pen-Tet-Mac-Car	1 (2.7)
AMP-AMX-TE-AMC-CTR	Pen-Tet- PenB -Cef	1 (2.7)
AMP-AMX-TE-AMC-CIP-E	Pen-Tet-PenB-Flu-Mac	3 (8.1)
AMP-AMX-TE-AMC-NA-E	Pen-Tet-PenB-Qui-Mac	1 (2.7)
AMP-AMX-TE-AMC-CTR-E	Pen-Tet-PenB-Cef-Mac	1 (2.7)
AMP-AMX-TE-AMC-CIP-E-MEM	Pen-Tet-PenB-Flu-Mac-Car	2 (5.4)
AMP-AMX-TE-AMC-CIP-NA-MEM	Pen-Tet-PenB-Flu-Qui-Car	1 (2.7)
AMP-AMX-TE-AMC-CIP-E-GEN	Pen-Tet-PenB-Flu-Mac-Ami	1 (2.7)
AMP-AMX-TE-AMC-CTR-CIP-E-MEM	Pen-Tet-PenB-Flu-Mac-Car	1 (2.7)
Total		37 (100.0)

^a^See Table 4 for definitions of abbreviations.

**Table 6:** MAR pattern and MARI of *Salmonella* Enteritidis (*n* = 37) isolated from raw chicken meat.

**Resistance pattern**	**No. of isolates (%)**	**No. of antibiotics**	**MARI**
AR-3	1 (2.70)	3	0.3
AR-4	14 (37.84)	4	0.4
AR-5	12 (32.43)	5	0.5
AR-6	5 (13.51)	6	0.6
AR-7	4 (10.81)	7	0.7
AR-8	1 (2.70)	8	0.8

Abbreviations: AR = antimicrobial resistance, AR-3 = AR against three antibiotics, AR-4 = AR against four antibiotics, AR-5 = AR against five antibiotics, AR-6 = AR against six antibiotics, AR-7 = AR against seven antibiotics, AR-8 = AR against eight antibiotics, MAR = multiple antibiotic resistance, MARI = multiple antibiotic resistance index.

**Table 7:** Tetracycline resistant (*tet*A) gene profile of the *Salmonella* species.

**Name of the isolates**	**No. of tested isolates**	**No. of positive isolates (%)**
*Salmonella* species	45	45 (100%)
*Salmonella* Enteritidis	37	37 (100%)

## Data Availability

The data that support the findings of this study are available on request from the corresponding author.
